# Identification and validation of an individualized prognostic signature of lung squamous cell carcinoma based on ferroptosis‐related genes

**DOI:** 10.1111/1759-7714.14195

**Published:** 2021-10-20

**Authors:** Xiayao Diao, Chao Guo, Lei Liu, Guige Wang, Shanqing Li

**Affiliations:** ^1^ Department of Thoracic Surgery, Peking Union Medical College Hospital Chinese Academy of Medical Sciences and Peking Union Medical College Beijing China

**Keywords:** ferroptosis, lung squamous cell carcinoma, overall survival, prognostic signature

## Abstract

**Background:**

Lung squamous cell carcinoma (LUSC), one of the main pathological types of lung cancer, has led to consequential socioeconomic burden. Ferroptosis is an iron‐dependent form of cell death process with potentials for therapeutic target in various kinds of tumors. However, whether ferroptosis‐related genes (FRGs) are associated with the prognosis of LUSC patients is still unclear. The aim of this study was to establish a FRGs‐based signature which could stratify patients with LUSC.

**Methods:**

The RNA sequencing profiles and corresponding clinical data of LUSC patients were retrieved from The Cancer Genome Atlas (TCGA) database and Gene Expression Omnibus (GEO) dataset. A FRG‐based signature was developed using the TCGA‐LUSC cohort and validated in the GEO cohort. Gene set enrichment analysis (GSEA) and analysis of immune cell characteristics were conducted to assess the relationship between FRGs and biological function or immune status. A nomogram based on selected clinical factors and the risk scores which were generated from the FRG‐based signature was developed using the TCGA cohort and validated in the GEO cohort.

**Results:**

A set of 16 FRGs, significantly associated with overall survival (OS) in the TCGA cohort, was identified and could classify LUSC patients into two risk groups. Kaplan–Meier analysis illustrated that the survival rate of the high‐risk group was significantly lower than the low‐risk group. Assessment and external validation of the signature showed that the survival predictive performance of this signature was adequate. Additionally, multiple pathways and functions were enriched through GSEA and the analysis of immune cell characteristics showed significantly different abundances of immune cells among the two risk groups. Finally, a nomogram integrating the FRG‐based signature and selected clinical factors was also developed and assessed in both the TCGA and GEO cohort.

**Conclusion:**

This study indicated the association between the FRGs and prognosis of patients with LUSC. Targeting ferroptosis may serve as a novel potential therapeutic alternative for LUSC.

## Graphical abstract

We developed a nomogram to predict the overall survival of lung squamous cell carcinoma patients using the risk score calculated from the ferroptosis‐related gene‐based signature and the T stage. The nomogram showed satisfactory discrimination power, calibration, and clinical usefulness in both the TCGA and GEO cohort.

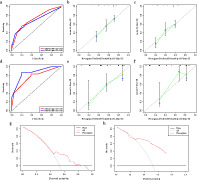

## INTRODUCTION

Lung cancer poses a severe global health problem with burdening medical and socioeconomic consequences, with about 2 093 870 new patients diagnosed and leading to about 1 761 000 deaths worldwide annually.^1^, ^2^ Non‐small cell lung cancer (NSCLC) represents the main subtype of lung cancer, accounting for about 85% of all lung cancer.^3^ NSCLC is also further classified into several histological types, including lung adenocarcinoma (LUAD), lung squamous cell carcinoma (LUSC), and large‐cell lung cancer (LCLC), as well as other infrequent types, among of which LUSC comprises 25%‐30% of all lung cancer cases.^4^ The clinical outcomes of lung cancer patients are related to associated risk factors and tumor stage at the time of diagnosis. Due to the lack of specific symptoms, more than two‐thirds of patients are diagnosed in advanced stages, leading to a 5‐year survival rate ranging from 13% to 1%.^5^, ^6^, ^7^ This phenomenon calls for early diagnosis and timely treatment.

In recent years, considerable developments have been achieved in targeted therapies, and several effective molecular targets such as epidermal growth factor receptor (EGFR) and anaplastic lymphoma kinase (ALK) have been identified.^8^ However, these targets are not efficient among LUSC patients, compared to LUAD because these two NSCLC subtypes have different mutation profiles.^9^, ^10^ Immune checkpoint inhibitors (nivolumab, pembrolizumab) in combination with paclitaxel and carboplatin are currently the first–line therapy for LUSC patients^11^, ^12^, ^13^ and treatment with immunotherapy can significantly improve the prognosis of patients.^14^ However, the strategy for immunotherapy is extremely expensive and side effects are also an inevitable issue, leading to unsatisfactory treatment outcomes for LUSC patients.^15^ Therefore, exploration of novel therapeutic mechanisms and development of effective prognostic models for accurate risk stratification and prognostic evaluation of LUSC patients are urgently needed.

Ferroptosis is an iron‐dependent form of necrotic cell death that is driven by the lethal accumulation of lipid peroxidation products and reactive oxygen species (ROS).^16^ The features and mechanisms of ferroptosis are different from those of typical cell death processes such as apoptosis and autophagy.^17^ In recent years, the induction of ferroptosis has been found to be a promising therapeutic alternative to trigger cancer cell death, especially for malignancies resistant to traditional therapy.^18^, ^19^ Emerging evidence, although limited, have shown that numerous genes are related to ferroptosis and play significant roles in the regulation of tumor progression in NSCLC. For instance, GPX4, which could play a pivotal role in the resistance to process of ferroptosis, was revealed to facilitate the proliferation of cancer cells of NSCLC.^20^ FSP1 and EGLN1 might act as suppressors to inhibit ferroptosis in lung cancer cells.^21^, ^22^ Upregulated NFS1 was found to be associated with resistance to ferroptosis in LUAD.^23^ Additionally, ferroptosis has also been reported to influence tumor progression by interacting with some immune cells.^24^, ^25^ All these discoveries have shown ferroptosis as a promising target for lung cancer treatment. Nevertheless, whether ferroptosis process and relevant ferroptosis‐related genes are associated with the prognosis of LUSC patients still requires further investigation.

In this present study, we collected the mRNA expression profiles of LUSC from public databases and identified the differentially expressed genes (DEGs) of LUSC which were also categorized as ferroptosis‐related genes (FRGs). Then, functional enrichment analysis was performed to explore the underlying mechanisms. Moreover, a prognostic multigene signature was constructed with these selected FRGs. Finally, a FRGs and clinical factors‐based model was constructed to improve the prognostic evaluation of LUSC patients. This study explored the potential prognostic value of ferroptosis‐related genes in LUSC and developed a user‐friendly tool to assess the risk and prognosis for patients with LUSC.

## METHODS

### Data collection

Two patient cohorts were included in this study. RNA sequencing profiles and corresponding clinical data of 502 patients with LUSC patients, including 502 tumor samples and 49 normal samples, were downloaded from The Cancer Genome Atlas (https://portal.gdc.cancer.gov) (TCGA cohort). The raw data of mRNA expression matrix and clinical information of 69 tumor samples were retrieved from the Gene Expression Omnibus (GEO) dataset (https://www.ncbi.nlm.nih.gov/geo/query/acc.cgi?acc=GSE73403) (GEO cohort).^26^ The platform of GSE73403 was GPL6480 (Agilent‐014850 Whole Human Genome Microarray 4x44K G4112F). Patients who met the following selection criteria were included: histologically diagnosed as LUSC, available gene expression data, and available prognostic data. In this study, the TCGA cohort was used as the training set and the GEO cohort as the validation set.

### Development and validation of the prognostic ferroptosis‐related gene signature

A total of 278 FRGs were gathered from the FerrDb database (http://www.zhounan.org/ferrdb/). We performed the following process to develop the prognostic signature. The differential analysis of the FRGs between LUSC and normal tissues were performed using the Wilcoxon test after within‐array replicate probes were replaced with their average via “limma” R package in the TCGA cohort.^27^, ^28^ The *p‐*value was adjusted with the false discovery rate (FDR).^29^ FDR < 0.05 and |log2 (FC)| ≥ 1 was considered statistically significant. To visualize the ferroptosis‐related DEGs, heatmap and volcano plot were generated using the “pheatmap” R package. Univariate Cox analysis of overall survival (OS) was performed to screen for FRGs with prognostic values. *p* < 0.05 were considered statistically significant. Subsequently, the least absolute shrinkage and selection operator (LASSO) regression algorithm was performed to establish the FRGs‐based signature.^30^, ^31^ LASSO regression analysis was conducted using the “glmnet” package in R software, Tenfold cross‐validation was utilized to filtrate candidate genes and identify the penalty parameter (λ), corresponding to the minimum value of partial likelihood deviance. The risk scores of each patient were calculated based on the expression level of selected FRGs and corresponding regression coefficients of genes. The prognostic risk score formula was constructed as follows:

$$\text{Risk score}=\sum_{i=1}^{n}\text{coefficients}*\text{Expression of FRGs}\left(i\right)$$

Using the risk score calculated based on this formula, the patients were divided into high‐ and low‐risk groups according to the median value of the risk score. We further evaluated the prognostic value of the ferroptosis‐related gene signature through Kaplan–Meier survival analysis. The “timeROC” package was utilized to perform time‐dependent receiver operating characteristic (ROC) curve analyses to evaluate the predictive discrimination of the FRGs‐based signature. Performance assessments of the signature were also conducted in the GEO cohort.

### Functional enrichment analysis

To investigate the potential molecular mechanisms of these FRGs which were significant in LASSO regression analysis, we performed gene ontology (GO) and Kyoto Encyclopedia of Genes and Genomes (KEGG) enrichment analyses of the FRGs among the high‐risk group and low‐risk group in the training set, which were selected according to the thresholds of |log2 (FC)| ≥ 1 and FDR < 0.05. The gene set enrichment analysis (GSEA) was conducted using the “clusterProfiler” R package. The pathways with *p* < 0.05 were significantly enriched.

### Analysis of immune cell characteristics

Utilizing the “CIBERSORT” R package, the proportions of 22 tumor‐infiltrating immune cells from each patient in the TCGA cohort were determined. In brief, CIBERSORT was performed to analyze the relative expression levels of 547 genes in individual tissue samples based on their gene expression profiles, to predict the abundance of 22 types of immune cells in each patient.^32^ Additionally, the enrichment levels for multiple immune cells of patients in the high‐ and low‐risk groups were compared to show the potential association between ferroptosis and immune status.

### Establishment and validation of the nomogram incorporating ferroptosis‐related gene signature

In this study, we developed a nomogram incorporating the risk score generated from the prognostic FRGs‐based signature and clinicopathological predictors in the training set using the “rms” R package. We then assessed the prognostic value of the nomogram through Kaplan–Meier survival analysis based on the high‐ and low‐risk groups stratified by the median value of the risk score, generated from the nomogram. The performance of the nomogram was also evaluated with respect to its discrimination and calibration in the training and validation set. The ROCs at 3‐ and 5‐year follow‐up were performed to assess the discrimination of the model, and calibration was evaluated by visualizing the discrepancy between actual probabilities and predicted probabilities using the calibration curves. In addition, the clinical usefulness of the nomogram was assessed by calculating the net benefits at different threshold probabilities in decision curve analysis (DCA). All these assessments were performed in both the TCGA cohort and the GEO cohort.

### Statistical analysis

All statistical analyses were performed using the R statistical software, version 4.0.2 (https://www.r-project.org). Continuous variables were analyzed using the Student's *t*‐tests, U tests, or nonparametric rank‐sum tests. Categorical variables were analyzed using the Chi‐squared tests or Fisher's exact tests. The OS between different risk groups was compared using the Kaplan–Meier analysis with the log‐rank test. Univariate Cox regression analysis and multivariate Cox regression analysis were implemented to identify independent prognostic predictors of OS. All statistical tests were two‐tailed, and *p* < 0.05 were considered statistically significant.

## RESULTS

### Patient cohort

**FIGURE 1:**
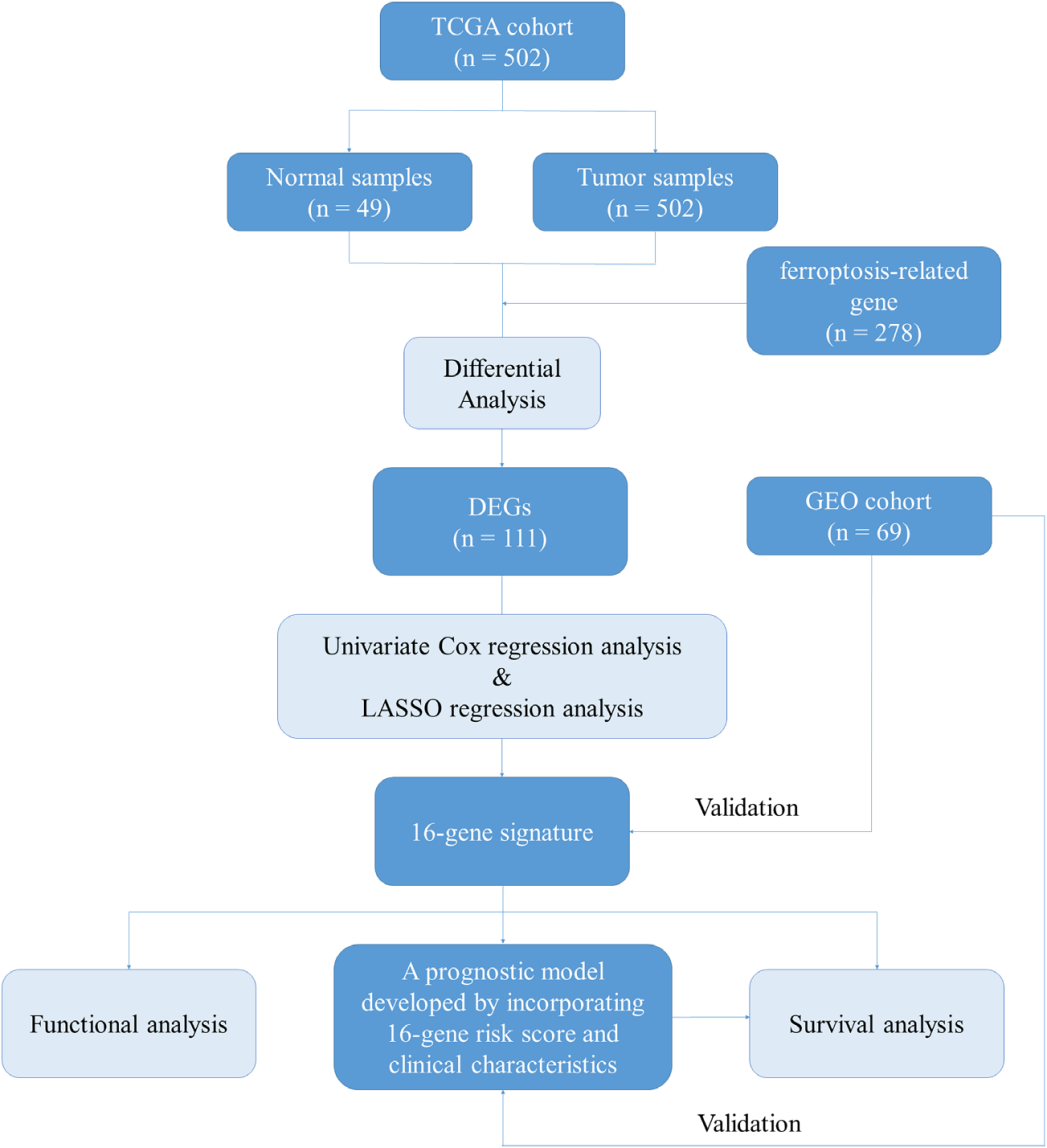
The flow chart of this study. TCGA, The Cancer Genome Atlas; GEO, gene expression omnibus; DEGs, differentially expressed genes

The flow diagram of this study is shown in Figure 1. A total of 502 patients with LUSC from the TCGA‐LUSC database and 69 patients with LUSC from the GEO dataset were finally included in this study.

### Identification of ferroptosis‐related DEGs in the TCGA cohort

**FIGURE 2:**
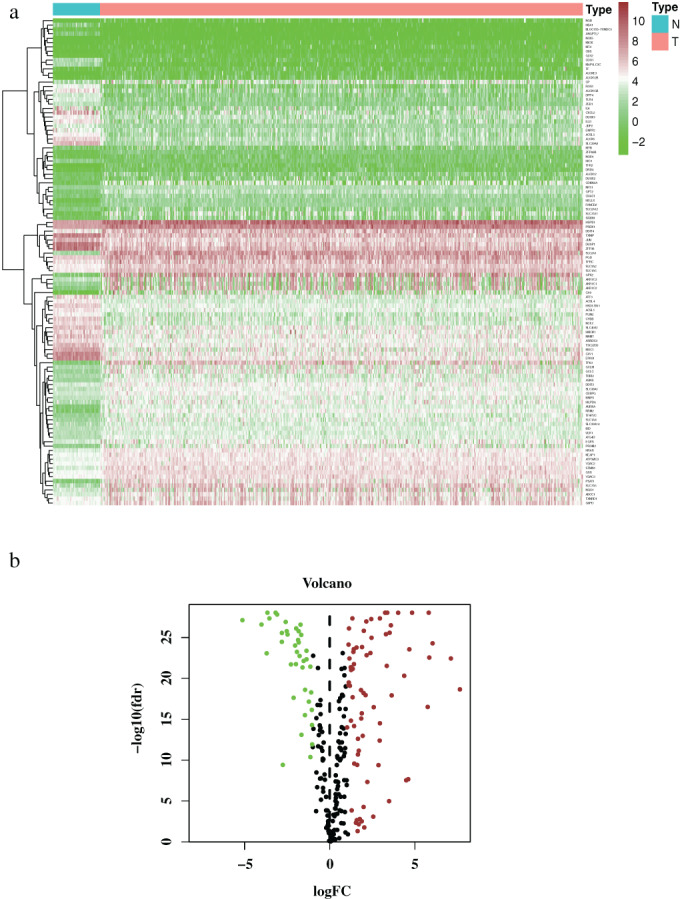
Differentially expressed ferroptosis‐related genes. (a) Heatmap and (b) Volcano plot

In total, 240 specific FRGs were identified with intersections of the transcription profile in the TCGA‐LUSC dataset and the FerrDb database, of which 155 genes were upregulated and 85 downregulated. Then, 111 ferroptosis‐related DEGs were identified based on the TCGA dataset, including 72 upregulated genes and 39 downregulated genes (Figure 2).

### Establishment and validation of the prognostic signature

**FIGURE 3:**
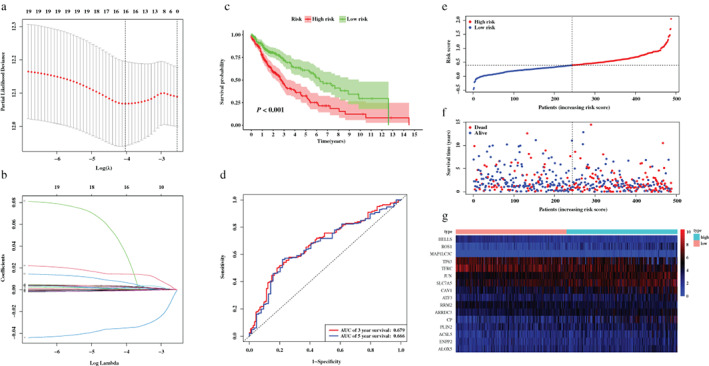
Development of the prognostic signature based on 16 ferroptosis‐related genes (FRGs) in the training set. (a,b) LASSO regression analysis identified 16 FRGs. (c) Survival analysis between risk groups defined by prognostic signature. (d) Time‐dependent receiver operator characteristic curve for predicting overall survival of the prognostic signature. (e) The distribution of risk score of each patient. (f) Survival statuses of patients in different groups. (g) Heatmap of expression profiles of FRGs incorporated in the signature

A total of 488 LUSC patients from the TCGA database were included in the training set. Based on the transcription profile of ferroptosis‐related DEGs, 20 FRGs were found associated with the prognosis of LUSC patients in univariate Cox regression and Table 1 demonstrates the general profile of survival associated FRGs in LUSC. Then, LASSO regression analysis was applied to establish a prognostic model using the expression profile of the 20 genes, and a 16‐gene signature was developed according to the optimal value of λ (Figure 3(a),(b)). After extracting the coefficient values, the risk score formula was presented in the format mentioned above. Consequently, a prognostic FRGs‐based signature for LUSC patients was established.

**TABLE 1:** The characteristics of ferroptosis‐related genes in LUSC (univariate Cox regression analysis)

Gene	HR	HR.95L	HR.95H	*p* ^a^
CP	1.005	1.001	1.010	0.011
CAV1	1.004	1.001	1.007	0.009
ATF3	1.007	1.001	1.013	0.025
HELLS	0.875	0.797	0.961	0.005
PLIN2	1.013	1.001	1.026	0.030
TFRC	0.999	0.997	1.000	0.036
RRM2	0.987	0.976	0.999	0.032
MUC1	1.003	1.000	1.006	0.022
ARRDC3	1.011	1.001	1.020	0.029
ACSL5	1.015	1.002	1.029	0.021
DUSP1	1.002	1.001	1.003	0.007
JUN	1.004	1.001	1.007	0.004
EPAS1	1.005	1.000	1.009	0.032
ROS1	1.040	1.012	1.06	0.004
MAP1LC3C	1.236	1.076	1.420	0.003
ENPP2	1.022	1.001	1.044	0.037
ALOX5	1.017	1.004	1.029	0.008
TP63	0.997	0.994	1.000	0.028
SLC39A8	1.020	1.006	1.035	0.006
SLC7A5	1.002	1.000	1.005	0.030

Abbreviations: HR, hazard ratio; LUSC, lung squamous cell carcinoma.

^a^ *p*‐values were obtained from the univariate Cox regression analysis of overall survival (OS) in The Cancer Genome Atlas (TCGA) cohort.

The risk score of each patient in the training set was calculated based on the formula, and patients were stratified into a low‐ or high‐risk group according to the determined median cutoff value (Figure 3(e)). Survival analysis showed that the survival rate of patients from the high‐risk group was significantly lower than the low‐risk group (*p* < 0.001, Figure 3(c)). The signature also showed an acceptable discrimination performance with AUCs of 0.679 and 0.666 at 3‐ and 5‐year follow‐up in the training set (Figure 3(d)). Figure 3(f) showed that an increase in risk score was associated with increasing number of patients had a risk of poor prognoses. In brief, patients in the high‐risk group were more likely to encounter death earlier. The expression levels of the FRGs which were included in signature were also performed in Figure 3(g).

**FIGURE 4:**
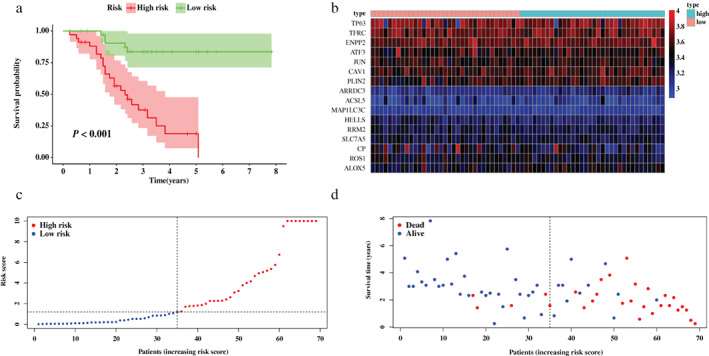
Validation of the prognostic signature based on 16 ferroptosis‐related genes (FRGs) in the validation set. (a) Survival analysis between risk groups defined by prognostic signature. (b) Heatmap of expression profiles of FRGs incorporated in the signature. (c) The distribution of risk score of each patient. (d) Survival statuses of patients in different groups

To validate the robustness of the signature developed from the training set, patients from the GEO cohort were also stratified into a high‐ or low‐risk group based on the median value generated from the same FRGs filtrated from the training set (Figure 4(c)). Similar to the results from the TCGA cohort, an obvious separation was shown in the Kaplan–Meier survival curve of the validation set (*p* < 0.001, Figure 4(a)). Further, Figure 4(b) illustrated similar expression levels of FRGs, compared to the TCGA cohort, and the risk plots also demonstrated remarkably different survival statuses between two risk groups (Figures 4(c),(d)).

### Gene set enrichment analysis

**FIGURE 5:**
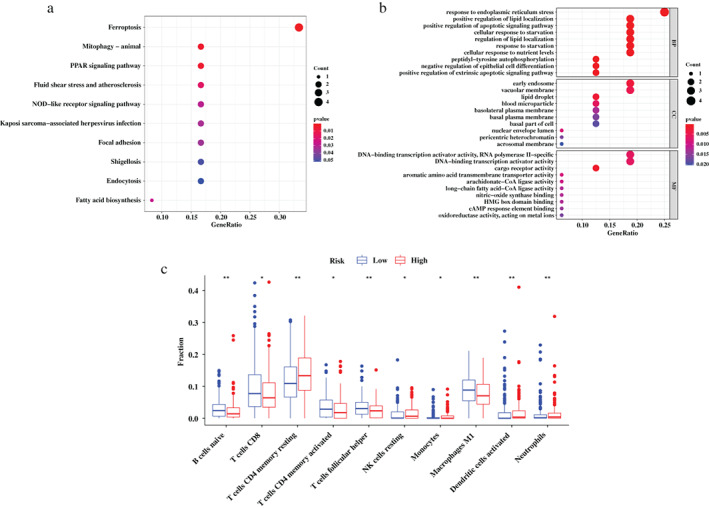
Gene set enrichment analysis (GSEA) analysis for the ferroptosis‐related genes (FRGs) incorporated in the prognostic signature and tumor‐infiltrating immune cells characteristics among risk groups defined by prognostic signature. (a) GSEA in Kyoto Encyclopedia of Genes and Genomes (KEGG) terms. (b) GSEA in gene ontology (GO) terms. (c) The difference of tumor‐infiltrating immune cells among risk groups as defined by the FRG‐based signature

The predictive power of the 16‐FRGs signature was associated with the biological function of these FRGs in LUSC. To explore the underlying mechanism, we conducted GSEA based on the selected FRGs incorporated in the signature to identify the enriched KEGG pathways and GO terms. KEGG terms (Figure 5(a)) mainly including ferroptosis, peroxisome proliferator‐activated receptors (PPAR) signaling pathway, fluid shear stress, and atherosclerosis as well as nucleotide‐binding oligomerization domain (NOD)‐like receptor signaling pathway were significantly enriched. Particularly, the enriched ferroptosis term was in consistency with the biological function of FRGs filtered by this study. As shown in Figure 5(b), GO enrichment analysis indicated enhanced activity of several biological processes or molecular functions such as response to endoplasmic reticulum stress, positive regulation of lipid localization, positive regulation of apoptotic signaling pathway, cellular response to starvation, DNA‐binding transcription activator activity, and cargo receptor activity.

### Difference of tumor infiltrating immune cells between risk groups

The discrepancies presented in tumor infiltrating immune cells in the TCGA cohort among the high‐ and low‐risk groups were explored to reveal the correlation between the tumor immune microenvironment and the FRGs‐based prognostic signature. The results illustrated that abundances of resting memory CD4+ T cells, resting natural killer (NK) cells, monocytes, activated dendritic cells, and neutrophils were significantly enriched (*p* < 0.05) in the high‐risk group compared to the low‐risk group. In contrast, the abundances of naive B cells, CD8+ T cells, activated memory CD4+ T cells, follicular helper T cells, and M1 macrophages in the high‐risk group were markedly lower than the low‐risk group (*p* < 0.05) (Figure 5(c)).

### Construction and performance assessment of the nomogram based on the ferroptosis‐related gene signature

**FIGURE 6:**
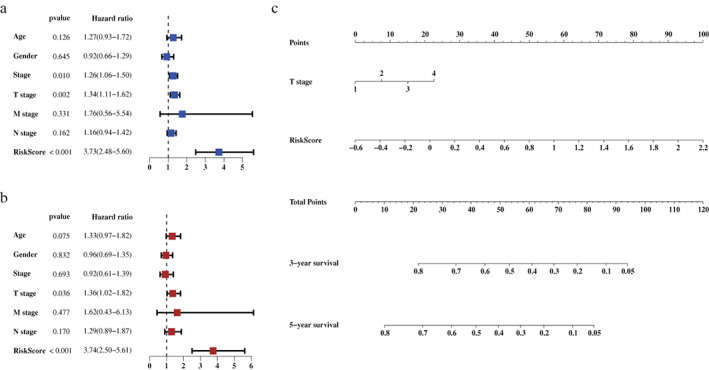
Construction of a nomogram based on the risk scores generated from the ferroptosis‐related gene signature and selected clinical factors. (a) Result of the univariate Cox regression analysis in the training set. (b) Result of the multivariate Cox regression analysis in the training set. (c) A nomogram based on the signature and clinical predictors

The risk score generated from the FRGs‐based signature and other clinical candidate predictors were tested using the univariate and multivariate Cox regression algorithm in the training set. All variables that were significant (*p* < 0.05) in the multivariate analysis were included in the model. Forest plots were performed to visualize the *p*‐value, confidence interval (CI), and hazard ratio (HR) generated from Cox regression analyses (Figure 6(a),(b)). Finally, we developed a nomogram to predict the 3‐ and 5‐year overall survival using the risk score calculated from the FRGs‐based signature and the T stage based on the American Joint Committee on Cancer (AJCC) TNM staging system (Figure 6(c)).

**FIGURE 7:**
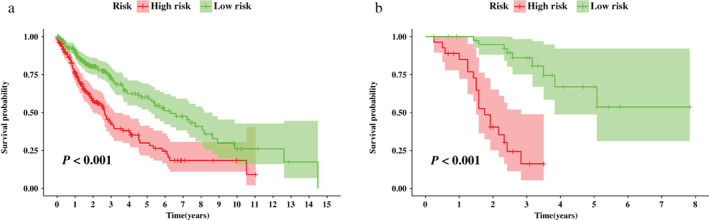
Survival analysis between risk groups stratified by the nomogram. (a) Survival analysis between nomogram‐defined risk groups in the training set. (b) Survival analysis between nomogram‐defined risk groups in the validation set

Based on the median risk score calculated from the nomogram, patients from the TCGA cohort were stratified into high‐ and low‐risk groups. Figure 7(a) indicated that patients in the high‐risk group had significantly shorter OS than those of the low‐risk group (*p* < 0.001), and was confirmed in the validation set (*p* < 0.001, Figure 7(b)).

**FIGURE 8:**
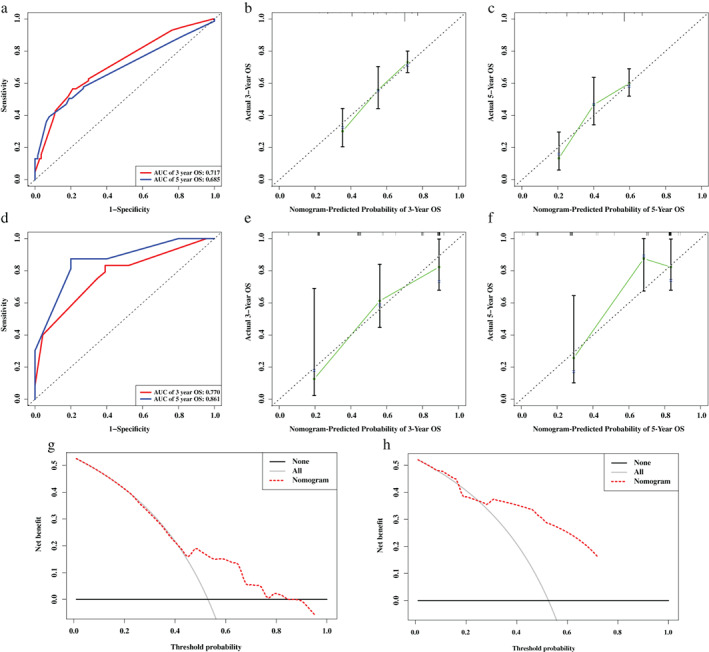
Assessment of a nomogram based on selected clinical factors and the risk scores generated from the ferroptosis‐related gene signature. (a) Time‐dependent receiver operating characteristic (ROC) curve for predicting overall survival (OS) of the nomogram in the training set. (b,c) Calibration curve evaluating the predictive accuracy of the nomogram in the training set at 3‐year survival (b) at 5‐year survival (c). (d) Time‐dependent ROC curve for predicting OS of the nomogram in the validation set. (e,f) calibration curve evaluating the predictive accuracy of the nomogram in the validation set at 3‐year survival (e) at 5‐year survival (f). (g) Decision curve analysis (DCA) evaluating the clinical usefulness of the nomogram in the training set. (h) DCA evaluating the clinical usefulness of the nomogram in the validation set

ROC analyses indicated adequate discrimination power with an AUC of 0.717, and 0.685 at 3‐ and 5‐year follow‐up (Figure 8(a)). Additionally, the calibration plot demonstrated good agreement between the model prediction and actual observation in the training set (Figure 8(b),(c)). Furthermore, the results of DCA showed that most part of the dashed curve was above the two solid lines (gray and black), demonstrating higher net benefits (Figure 8(g)). In other words, the nomogram demonstrated promising value as a clinical decision‐making tool. Likewise, this prognostic model also showed satisfying discrimination, calibration, and clinical usefulness in the GEO cohort (Figure 8(d)–(f),(h)).

## DISCUSSION

Over the past several years, ferroptosis has gained considerable attention from researchers. This newly discovered type of cell death process is characterized by the excessive accumulation of iron‐dependent lipid hydroperoxides.^17^ Dysregulation in iron hemostasis leads to excessive iron accumulation in cells and may activate ferroptosis.^33^ Over these years, this unique pattern of programmed cell death has become the focus of numerous studies and increasing evidence has illustrated that this process is highly recognized as a promising therapeutic strategy for various kinds of cancer types, including ovarian cancer and hepatocellular carcinoma.^34^, ^35^ However, limited studies have focused on the specific role of ferroptosis in LUSC as well as its potential mechanism and biological function.

In the present study, 278 ferroptosis‐related genes and two mRNA expression profiles of LUSC patients (TCGA‐LUSC and GSE73403) were identified. In the TCGA cohort, 111 ferroptosis‐related DEGs were identified between LUSC samples and normal samples. Then, univariate Cox regression analysis indicated that there were 20 genes associated with OS among the DEGs, and LASSO regression finally identified 16 FRGs (CP, CAV1, ATF3, HELLS, PLIN2, TFRC, RRM2, ARRDC3, ACSL5, JUN, ROS1, MAP1LC3C, ENPP2, ALOX5, TP63, and SLC7A5) which were used to develop the ferroptosis‐related gene signature. The potential mechanism of these FRGs were diverse, for example, depletion of CP promoted erastin‐ and RSL3‐induced ferroptosis.^36^ Overexpression of CAV1 led to augmented ferroptosis susceptibility through high extracellular regulated kinases (ERK) pathway activation.^37^ Knockout of ATF3 suppressed erastin‐induced ferroptosis and lipid peroxidation.^38^ HELLS inhibited ferroptosis by activating lipid metabolism‐associated genes.^22^ Knockdown of PLIN2 facilitated ALOX15 higher expression and acceleration of ferroptosis.^39^ Upregulation of TRFC promoted ferroptosis by increasing the intracellular iron load.^40^ RRM2, JUN, and TP63 were found to increase cellular resistance against ferroptosis by regulating the synthesis, utilization, and regeneration of glutathione.^41^, ^42^, ^43^ A previous study indicated that overexpression of ENPP2 could inhibit erastin‐induced ferroptosis.^44^ ALOX5 led to ferroptosis through mediating lipid peroxidation.^45^ Solute carrier family 7 member 5 (SLC7A5) belongs to the solute carrier family, which could induce ferroptosis by selective oxidation of esterified phosphatidylethanolamines.^46^ Subsequent Kaplan–Meier survival analyses verified the prognostic value of the 16 FRGs signature in the training set and validation set. The survival rates between the two risk groups stratified according to the prognostic signature were remarkably different. Moreover, the signature also showed adequate discriminatory accuracy for patients with LUSC.

To explore the potential mechanisms by which the FRGs‐based signature effectively stratifies LUSC patients, GSEA analysis demonstrated significant activity of multiple pathways mediated by FRGs incorporated in the signature. KEGG pathway analysis illustrated that FRGs were significantly enriched in the ferroptosis, PPAR signaling pathway, fluid shear stress, and atherosclerosis as well as NOD‐like receptor signaling pathway, with the exception of mitophagy in animals. Ferroptosis is recognized as a novel treatment strategy to inhibit tumor development in various tumors including lung cancer. Because of the association between ferroptosis regulators and chemotherapy resistance or immunotherapeutic effects, it is reasonable to utilize ferroptosis‐related drugs to assist tumor therapy. The FRGs identified in this study may serve as promising targets in treating patients with solid malignant tumors.^47^ Additionally, PPARs involve in specific biological functions such as metabolic homeostasis, differentiation, cell proliferation, and apoptosis.^48^ PPAR‐γ ligands could inhibit tumor growth and induce apoptosis of lung cancer cells.^49^ Furthermore, NOD‐like receptors may play an important role in the innate immune system and are considered as promising targets in cancer immunotherapy.^50^ Moreover, GO analysis of these selected FRGs showed that genes were mainly enriched in the following biological functions: response to endoplasmic reticulum stress, positive regulation of lipid localization, positive regulation of apoptotic signaling pathway, cellular response to starvation, DNA‐binding transcription activator activity, and cargo receptor activity. Despite the numerous evidence of ongoing endoplasmic reticulum stress in many forms of cancer, whether these processes ultimately inhibit or promote tumor growth in patients remains a field of further research.^51^ Besides, localization and accumulation of lipid droplets in cancers suggest related organelles as potential targets for cancer therapy and are also associated with a poor outcome in cancer patients.^52^, ^53^

When investigating the correlation of the FRGs‐based signature with the immune status of tumors, we found that the abundances of naive B cells, CD8+ T cells, activated memory CD4+ T cells, follicular helper T cells, and M1 macrophages were significantly lower in the high‐risk group compared to the low‐risk group, suggesting that the LUSC patients in the high‐risk group may have worse immune function and immune status. A previous study indicated that CD8+ T cells could induce ferroptosis in tumor cells, and the immune system can regulate ferroptosis susceptibility to suppress tumorigenesis.^25^ Broderick et al. also revealed that memory CD4+ T cells which constitutively present in the microenvironment of lung cancer could be mobilized by IL‐12 to proliferate and kill tumor cells.^54^ Furthermore, since M1 macrophages may possess inflammatory and anti‐tumorigenic features, high abundance of M1 macrophages in tumor could indicate increase OS in patients with NSCLC.^55^ In general, the dysregulation of tumor immune microenvironment may take responsibility for the discrepancy in survival prognosis among the risk groups identified by the prognostic FRGs‐based signature.

In the present study, we also developed a nomogram based on selected clinical factors and the risk scores which were generated from FRGs‐based signature to better predict prognosis in LUSC patients. The performance of this model was evaluated and externally validated. To our best knowledge, this has been the first study that developed FRGs‐based signature and nomogram to predict prognosis in LUSC. However, several limitations in our study should be considered. First, it was a retrospective study based on the data from public databases, some information may be unavailable. Second, the molecular mechanisms of LUSC could not be fully demonstrated without in vitro and in vivo experiments. Therefore, further research is urgently warranted to validate these findings.

In conclusion, a signature based on ferroptosis‐related genes was constructed and provide potential biomarkers for prognostic prediction in LUSC. The treatment targeting ferroptosis might be a novel promising therapeutic alternative for patients with LUSC.

## CONFLICT OF INTEREST

The authors declare that they have no competing interests.
